# Metabolic Side-Effects of the Novel Second-Generation Antipsychotic Drugs Asenapine and Iloperidone: A Comparison with Olanzapine

**DOI:** 10.1371/journal.pone.0053459

**Published:** 2013-01-09

**Authors:** Heidi N. Boyda, Ric M. Procyshyn, Catherine C. Y. Pang, Erin Hawkes, Daniel Wong, Chen Helen Jin, William G. Honer, Alasdair M. Barr

**Affiliations:** 1 Department of Anesthesiology, Pharmacology and Therapeutics, University of British Columbia, Vancouver, British Columbia, Canada; 2 Department of Psychiatry, University of British Columbia, Vancouver, British Columbia, Canada; 3 British Columbia Mental Health and Addictions Services, British Columbia Mental Health and Addictions Research Institute, Vancouver, British Columbia, Canada; Omaha Veterans Affairs Medical Center, United States of America

## Abstract

**Background:**

The second generation antipsychotic (SGA) drugs are widely used in psychiatry due to their clinical efficacy and low incidence of neurological side-effects. However, many drugs in this class cause deleterious metabolic side-effects. Animal models accurately predict metabolic side-effects for SGAs with known clinical metabolic liability. We therefore used preclinical models to evaluate the metabolic side-effects of glucose intolerance and insulin resistance with the novel SGAs asenapine and iloperidone for the first time. Olanzapine was used as a comparator.

**Methods:**

Adults female rats were treated with asenapine (0.01, 0.05, 0.1, 0.5, 1.0 mg/kg), iloperidone (0.03, 0.5, 1.0, 5.0, 10.0 mg/kg) or olanzapine (0.1, 0.5, 1.5, 5.0, 10.0 mg/kg) and subjected to the glucose tolerance test (GTT). Separate groups of rats were treated with asenapine (0.1 and 1.0 mg/kg), iloperidone (1.0 and 10 mg/kg) or olanzapine (1.5 and 15 mg/kg) and tested for insulin resistance with the hyperinsulinemic-euglycemic clamp (HIEC).

**Results:**

Asenapine showed no metabolic effects at any dose in either test. Iloperidone caused large and significant glucose intolerance with the three highest doses in the GTT, and insulin resistance with both doses in the HIEC. Olanzapine caused significant glucose intolerance with the three highest doses in the GTT, and insulin resistance with the higher dose in the HIEC.

**Conclusions:**

In preclinical models, asenapine shows negligible metabolic liability. By contrast, iloperidone exhibits substantial metabolic liability, comparable to olanzapine. These results emphasize the need for appropriate metabolic testing in patients treated with novel SGAs where current clinical data do not exist.

## Introduction

The second generation antipsychotic (SGA) drugs represent the preferred choice of pharmacological treatment for schizophrenia. Many SGAs are also approved for additional indications, and are widely used off-label for other psychiatric disorders [1]. Despite the lower incidence of extrapyramidal side-effects with SGAs compared to their predecessors, over the last decade it has become clear that the majority of SGAs are associated with substantial unwanted side-effects. These side-effects are predominantly metabolic disturbances which include the components of metabolic syndrome [2]–[5]: weight-gain, adiposity, hyperlipidemia, glucose intolerance and insulin resistance. Pharmacological interventions, such as anti-diabetic drugs, may be only partly successful in reversing metabolic changes [6], [7], leaving those with SGA drug-induced metabolic side-effects at substantially increased risk of developing cardiovascular disease and Type 2 Diabetes Mellitus (DM) [8], [9], resulting in increased morbidity and mortality.

However, within the SGA class, there is considerable variation with regards to metabolic liability [10]–[12]. This has been most commonly assessed by weight-gain. Numerous studies have confirmed a continuum of metabolic liability, with the drugs clozapine and olanzapine causing greatest weight-gain, through intermediate effects with drugs including risperidone and quetiapine, to least weight-gain with ziprasidone and aripiprazole. Importantly, even the latter drugs are not body-weight-neutral, as weight-gain is greater than with placebo [13], particularly in patients taking antipsychotic drugs for the first time [14]. Fewer studies have examined the effects of SGAs on glucose control and insulin resistance, likely due to the greater difficulty of such measurements. Nevertheless, the pattern resembles that for weight-gain, with greatest insulin resistance caused by clozapine and olanzapine, and least by ziprasidone and aripiprazole [15]. Considerable evidence indicates that the effects of SGAs on insulin resistance and other metabolic indices, such as dyslipidemia [16], are not simply due to drug-induced weight-gain. Studies have reported new-onset diabetes in the absence of obesity or substantial weight-gain in SGA-treated patients [17], while acute effects of SGAs in non-psychiatric subjects included rapid-onset glucose intolerance in the absence of major weight-gain [18]–[20].

The metabolic side-effects of SGAs have been modeled in preclinical rodent paradigms [21], using sophisticated techniques such as the glucose tolerance test and hyperinsulinemic-euglycemic clamp [22]. Acute and chronic studies of SGA-induced insulin resistance and glucose intolerance have largely replicated clinical findings. Furthermore, the animal models are strongly homologous with clinical data, as drugs with greater metabolic liability in humans exert stronger metabolic effects in the preclinical paradigms [16], [23]–[31]. These models are therefore useful not only in helping to understand the biological basis of metabolic side-effects, but also in predicting such effects in novel antipsychotic drugs.

The two novel SGA drugs asenapine and iloperidone were both approved by the US Food and Drug Administration in 2009 for the treatment of schizophrenia [32]. While the clinical efficacy of these new drugs compared to placebo has been confirmed in registration trials, little is known about the metabolic side-effects of these compounds [12]. Some data are published regarding weight-gain, but to our knowledge, there has been no reported documentation of glucose intolerance and insulin resistance with these drugs, which are the core symptoms of Type 2 DM. Given the utility of preclinical models in predicting metabolic dysregulation caused by SGAs, we conducted a study of both glucose tolerance and insulin resistance with asenapine and iloperidone, using established procedures, across a wide range of doses. For reference, we concurrently measured metabolic dysregulation in animals treated with olanzapine, as this drug is known to reliably cause metabolic dysregulation.

## Materials and Methods

### Animals

Adult female Sprague-Dawley rats (250–275 g) from Charles River (Montreal, Canada) were habituated to the UBC colony for one week. Females are the preferred sex for rodent models of antipsychotic drug-induced metabolic dysregulation because they exhibit more consistent metabolic abnormalities than males [21], [33], [34]. Rats were pair-housed and maintained on a 12-hour light-dark cycle (lights on at 07∶00hours) under ambient temperature (22±1°C), with food and water available *ad libitum*. Approval by the UBC Animal Care and Use Committee was established for all procedures; animals were treated in accordance with the NIH Guidelines for the Care and Use of Laboratory Animals [35].

### Pharmacological Agents and Solutions

Antipsychotic drugs included asenapine [Sigma-Aldrich Inc., St. Louis, MO,], iloperidone and olanzapine [Toronto Research Chemicals Inc., Toronto, ON]. All dosing solutions were prepared daily. Asenapine was formulated in a vehicle composed of 0.9% saline with the addition of 10 µL 1 M hydrochloric acid; iloperidone and olanzapine were formulated in 50% polyethylene glycol 400, 40% distilled water and 10% ethanol (PEG solution) [36]. Antipsychotics were dissolved in a volume of 1 ml/kg. For clamp experiments, recombinant human insulin (Humulin R) [Eli Lily, Indianapolis, IN) and dextrose (50%) were formulated in 0.9% w/v saline. All other chemicals were of reagent grade.

### Intraperitoneal Glucose Tolerance Test (IGTT) (see Fig. 1A for Sequence of Events)

All rats were given a baseline IGTT prior to drug administration, as described previously [26]. Briefly, animals were fasted overnight for 16±2 hours; the following morning animals were wrapped in a towel to minimize stress, and a drop of saphenous venous blood was procured with a 25-gauge needle for baseline blood glucose measurement. Subsequently, animals received a glucose challenge (1 g/kg/ml, i.p.) followed by repeated sampling of blood glucose readings every 15 min for two hours. Glucose measurements were determined by handheld glucometer (One Touch Ultra). Based on total glucose levels, rats were rank ordered and randomly matched to one of six treatment groups (vehicle or five different doses of asenapine [n = 8–9 per group]) the following week. There was always one week minimum duration between subsequent IGTTs.

**Figure 1:**
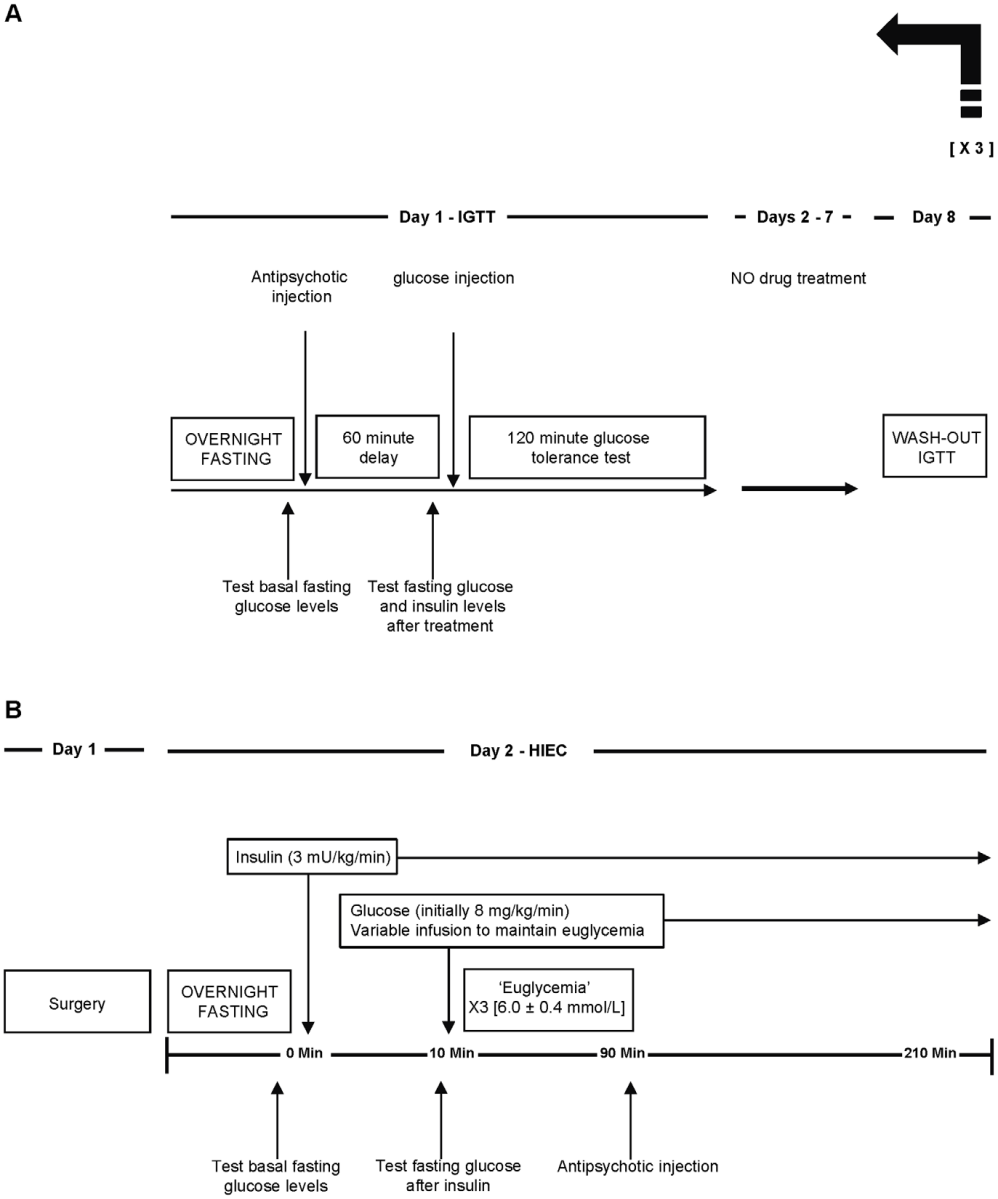
Experimental protocol. Describing (**A**) the intraperitoneal glucose tolerance test and (**B**) the hyperinsulinemic-euglycemic clamp with acute antipsychotic drug treatment.

For the asenapine IGTT, rats were fasted as above. Baseline blood glucose levels were measured, and then a single dose of asenapine (0.01, 0.05, 0.1, 0.5 or 1.0 mg/kg) or vehicle was administered by s.c. injection; each rat received only one dose. A second measurement of blood glucose was taken 30 minutes later, to assess drug treatment on fasting glucose levels. Subsequently, a saphenous blood draw using heparinized collecting tubes was performed to obtain plasma samples for analysis of insulin levels; extracted blood samples were centrifuged (10,000 RPM, 10 Min, 4°C) and samples stored at −80°C. Immediately afterwards, the IGTT commenced, whereby animals received a challenge injection of 1 g/ml/kg of glucose. Glucose levels were monitored and recorded every 15 minutes for two hours duration. Animal handlers were blinded to drug treatment. One week after the asenapine IGTT, rats received another baseline IGTT, for re-randomization for treatment with iloperidone the following week. Rats received either vehicle or iloperidone (0.03, 0.5, 1.0, 5.0 or 10.0 mg/kg). Following another baseline IGTT and re-randomization, rats received the final antipsychotic treatment with vehicle or olanzapine (0.1, 0.5, 1.5, 5.0 or 10.0 mg/kg).

### Surgical Preparations for Hyperinsulinemic-Euglycemic Clamp (HIEC)

Rats were prepared for surgery under isoflurane anesthesia and pre-operative ketoprofen (5 mg/kg, s.c). Heparinized saline-filled polyethylene cannulae (PE50) were inserted into the right common carotid artery and both exterior jugular veins. The arterial cannula was used to sample blood for measurement of glucose levels and venous cannulae were used for the infusion of insulin and dextrose. All cannulae were tunneled subcutaneously to the nape of neck and exteriorized. Animals recovered for 24 hours prior to the HIEC: two animals did not make a full recovery and were excluded.

### HIEC Procedures (see Fig. 1B for Sequence of Events)

Overnight fasted rats (16±2 hours) were habituated to the cage prior. The two venous cannulae were connected to auxiliary heparinized saline-filled PE50 tubing, directly attached to infusion-only pumps (Harvard Apparatus, Holliston, MA). After a baseline blood glucose reading from the arterial cannula, insulin infusion (3 mU/kg/min) was initiated (t = 0 min) and kept running at a constant rate for the entire experiment. Dextrose (50% w/v) infusion commenced at 8 mg/kg/min (0.96 ml/kg/hr) at t = 10 min and the glucose infusion rate (GIR) was adjusted as needed, every 10 minutes, to maintain glucose concentrations at 6.0 mmol/L. Euglycemia was determined when three consecutive blood glucose measurements presented 6.0±0.4 mmol/L at the same GIR. Animals then randomly received a single s.c injection of either vehicle or asenapine (0.1, 1.0 mg/kg), iloperidone (1.0, 10.0 mg/kg) or olanzapine (1.5, 15.0 mg/kg) [n = 5–7 per group], and the clamp was continued for 120 minute duration. The sample size of n = 5–7 animals per group is consistent with previously published studies [27], [28]. Handlers were blinded to drug treatment.

### Plasma Insulin Measurement by ELISA

Insulin levels were measured using the ultra-sensitive rat insulin ELISA kit (Crystal Chem Inc., IL, USA). Plasma samples (5 µl) were added and analyzed, in duplicate, on 96 well plates. Samples were incubated, followed by repeated washes. Substrate was added and absorbance measured at 450 nm –630 nm, as previously [37], [38]. Calibrators were prepared and used to generate a calibration curve. A reference (non-fasted) animal’s plasma added to all plates served as reference standard; confirming a high intra-plate reliability, with mean run-to-run correlations of 0.996 (range 0.994–0.999).

### Insulin Resistance

Determination of insulin resistance in rats was accomplished using the homeostatic model assessment of insulin resistance (HOMA-IR) equation (1) [39]. The product of both the fasting levels of glucose (expressed as mmol/L) and insulin (µU/ml) 30 minutes post-drug administration is divided by a constant of 22.5. Greater insulin resistance is represented via a larger calculated HOMA-IR score.
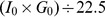
(1)where I_0_ and G_0_ are fasting insulinemia and glycaemia.

### Statistical Analysis

Metabolic indices during the IGTT were analyzed by one-way analysis of variance (ANOVA), with drug dose as the between group factor. For the IGTT, glucose data were summed as the area-under-the-curve throughout the 120 minute procedure [36]. For the HIEC data, all drugs were included in the overall ANOVA, as the same vehicle group was use for all between-drug comparisons. Alpha value was set at *p*<0.05. LSD post-hoc tests were conducted when a main effect or interaction between main effects was significant. Data were analyzed with SPSS software, Chicago, IL, version 20.

## Results

### IGTT

**Figure 2:**
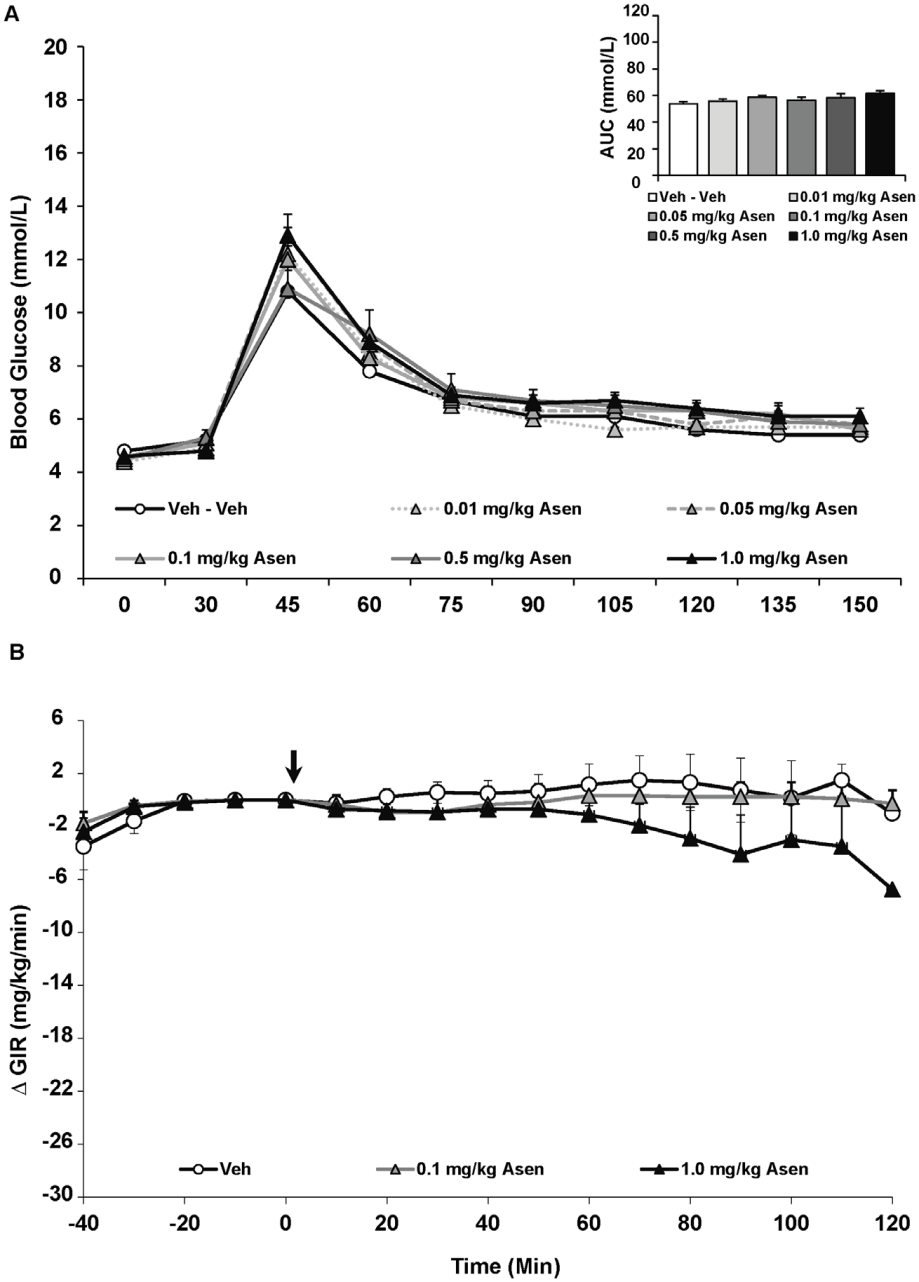
Acute effects of the antipsychotic drug asenapine on glucose levels in adult female rats. (**A**) Animals (n = 8–10 per group) received a single injection of vehicle or asenapine (0.01, 0.05, 0.1, 0.5, 1 mg/kg, s.c). Glucose levels were recorded prior to drug treatment in overnight-fasted rats at Time 0, and then 30 minutes following drug administration (*x-axis*). Immediately following this glucose measurement, all rats were subjected to a glucose tolerance test by receiving an intraperitoneal challenge injection of 1 mg/ml/kg of glucose, and blood glucose levels were measured every 15 minutes for the next two hours. Total cumulative glucose levels for each treatment group are summed as the “area under the curve” during the glucose tolerance test by graph inset (*top right*). Values represent group means ± SEM. (**B**) A separate cohort of animals (n = 6–8 per group) were fasted overnight and subjected to the hyperinsulinemic-euglycemic clamp. After animals reached euglycemia (three consecutive blood glucose readings of 6.0±0.4 mmol/L), rats were treated with vehicle, low (0.1 mg/kg) or high dose (1.0 mg/kg) asenapine (arrow at t = 0 min). Glucose levels were recorded every 10 minutes and the glucose infusion rate was adjusted as needed. Glucose infusion rates for each treatment group are presented as change in glucose infusion rate from euglycemia. Values represent group means ± SEM.

Analysis of the IGTT with asenapine revealed no significant effect of drug treatment on fasting glucose levels or glucose intolerance following glucose challenge (Figure 2A). Interestingly, asenapine affected fasting insulin levels [F_(5,43) = _3.03, p<0.05], whereby the three lower doses of the drug *decreased* insulin levels compared to controls, although this was only significant for the lowest asenapine dose (p<0.05) (Table 1). Similarly, asenapine significantly affected insulin resistance [F_(5,43) = _2.59, p<0.05], measured by HOMA-IR, as insulin resistance was significantly reduced with the lowest asenapine dose (0.01 mg/kg) and a strong trend with the next two doses (0.05 and 0.1 mg/kg) (Table 1).

**Table 1:** Mean concentration of fasting glucose, insulin and HOMA-IR scores in antipsychotic drug treated rats.

Antipsychotic Drug	Measure	Treatment Dose (mg/kg)					
**Asenapine**		**0**	**0.01**	**0.05**	**0.1**	**0.5**	**1.0**
	**G_0_**	5.1±0.3	4.9±0.3	5.1±0.2	5.0±0.1	5.4±0.4	4.8±0.2
	**I_0_**	24.6±5.7	16.3±2.2*	21.7±3.0	20.4±3.1	29.7±4.4	33.3±3.5
	**HOMA-IR**	5.7±1.5	3.4±0.4*	4.9±0.7	4.5±0.7	7.2±1.3	7.2±1.0

**Iloperidone**		**0**	**0.03**	**0.5**	**1.0**	**5.0**	**10.0**
	**G_0_**	4.6±0.2	4.9±0.2	4.6±0.2	4.9±0.2	5.1±0.2	5.2±0.3
	**I_0_**	21.6±3.6	21.6±2.6	28.2±5.0	40.1±4.9*	44.9±3.6*	58.8±7.7*
	**HOMA-IR**	4.6±0.8	4.7±0.5	5.9±1.2	8.8±1.2*	10.2±0.8*	13.8±2.2*

**Olanzapine**		**0**	**0.1**	**0.5**	**1.5**	**5.0**	**15.0**
	**G_0_**	5.0±0.2	5.3±0.2	5.3±0.3	5.7±0.3	5.2±0.2	5.0±0.2
	**I_0_**	19.7±1.7	32.5±6.0	35.9±5.7	36.3±2.7	41.4±6.6*	47.8±5.8*
	**HOMA-IR**	4.5±0.5	7.8±1.5	8.7±1.6	9.2±1.1	9.6±1.5	10.8±1.5

I_0_ = fasting insulin levels (µU/ml); G_0_ =  fasting glucose levels (mmol/L); HOMA-IR = homeostasis model assessment of insulin resistance (µU⋅mmol)/(ml⋅L).

Rats were treated with five separate doses of asenapine, iloperidone, olanzapine or vehicle. Values represented as means ± SEM at t = 30 min during the IGTT.

* indicates different from vehicle-treated animals, p<0.05.

**Figure 3:**
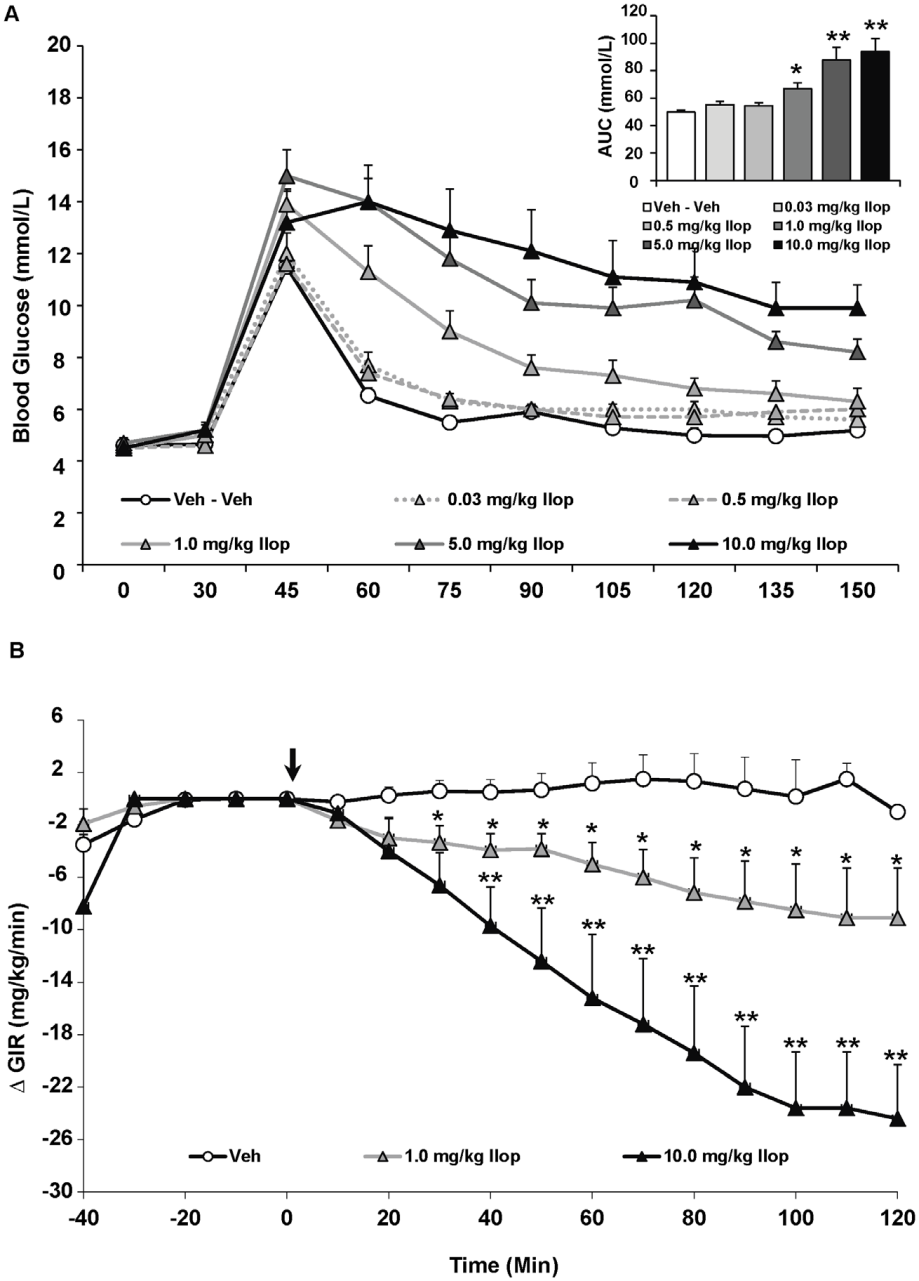
Acute effects of the atypical antipsychotic drug iloperidone on glucose levels in adult female rats. (**A**) Animals (n = 8–10 per group) received a single injection of vehicle or iloperidone (0.03, 0.5, 1.0, 5.0, 10.0 mg/kg, s.c). Glucose levels were recorded prior to drug treatment in overnight-fasted rats at Time 0, and then 30 minutes following drug administration (*x-axis*). Immediately following this glucose measurement, all rats were subjected to a glucose tolerance test by receiving an intraperitoneal challenge injection of 1 mg/ml/kg of glucose, and blood glucose levels were measured every 15 minutes for the next two hours. Total cumulative glucose levels for each treatment group are summed as the “area under the curve” during the glucose tolerance test by graph inset (*top right*). Values represent group means ± SEM. * indicates different from vehicle-treated animals, p<0.05; ** indicates different from vehicle and 0.03–1.0 mg/kg iloperidone-treated animals, p<0.01 (**B**). A separate cohort of animals (n = 6–8 per group) were fasted overnight and subjected to the hyperinsulinemic-euglycemic clamp. After animals reached euglycemia (three consecutive blood glucose readings of 6.0±0.4 mmol/L), rats were treated with vehicle, low (1.0 mg/kg) or high dose (10.0 mg/kg) iloperidone (arrow at t = 0 min). Glucose levels were recorded every 10 minutes and the glucose infusion rate was adjusted as needed. Glucose infusion rates for each treatment group are presented as change in glucose infusion rate from euglycemia Values represent group means ± SEM. * indicates different from vehicle-treated animals, p<0.05; ** indicates different from vehicle and 1.0 mg/kg iloperidone-treated animals, p<0.05.

By contrast, while iloperidone had no effect on fasting glucose levels, it exhibited a strongly significant effect on glucose tolerance during the IGTT [F_(5,43) = _13.06, p<0.0001] (Figure 3A). Post-hoc analysis revealed a dose-dependent effect whereby the three highest doses of iloperidone (1.0, 5.0 and 10.0 mg/kg) increasingly elevated glucose intolerance compared to controls (p<0.05), with the two highest doses causing increased glucose intolerance compared to both controls and the three lower doses of the drug (p<0.001). There was manifest as a striking increase of 88% and 91% in glucose levels with the two highest doses of iloperidone compared to vehicle-treated rats. Regarding insulin, the ANOVA indicated a main effect of drug on fasting insulin levels [F_(5,43) = _8.47, p<0.0001] (Table 1), reflecting a dose-dependent elevation of insulin levels. Insulin was significantly higher than controls with the three highest doses of iloperidone (p<0.05). Similarly, HOMA-IR values also significantly increased by drug treatment [F_(5,43) = _7.90, p<0.0001] (Table 1), as the three highest doses of iloperidone induced greater insulin resistance compared to controls (p<0.01).

**Figure 4:**
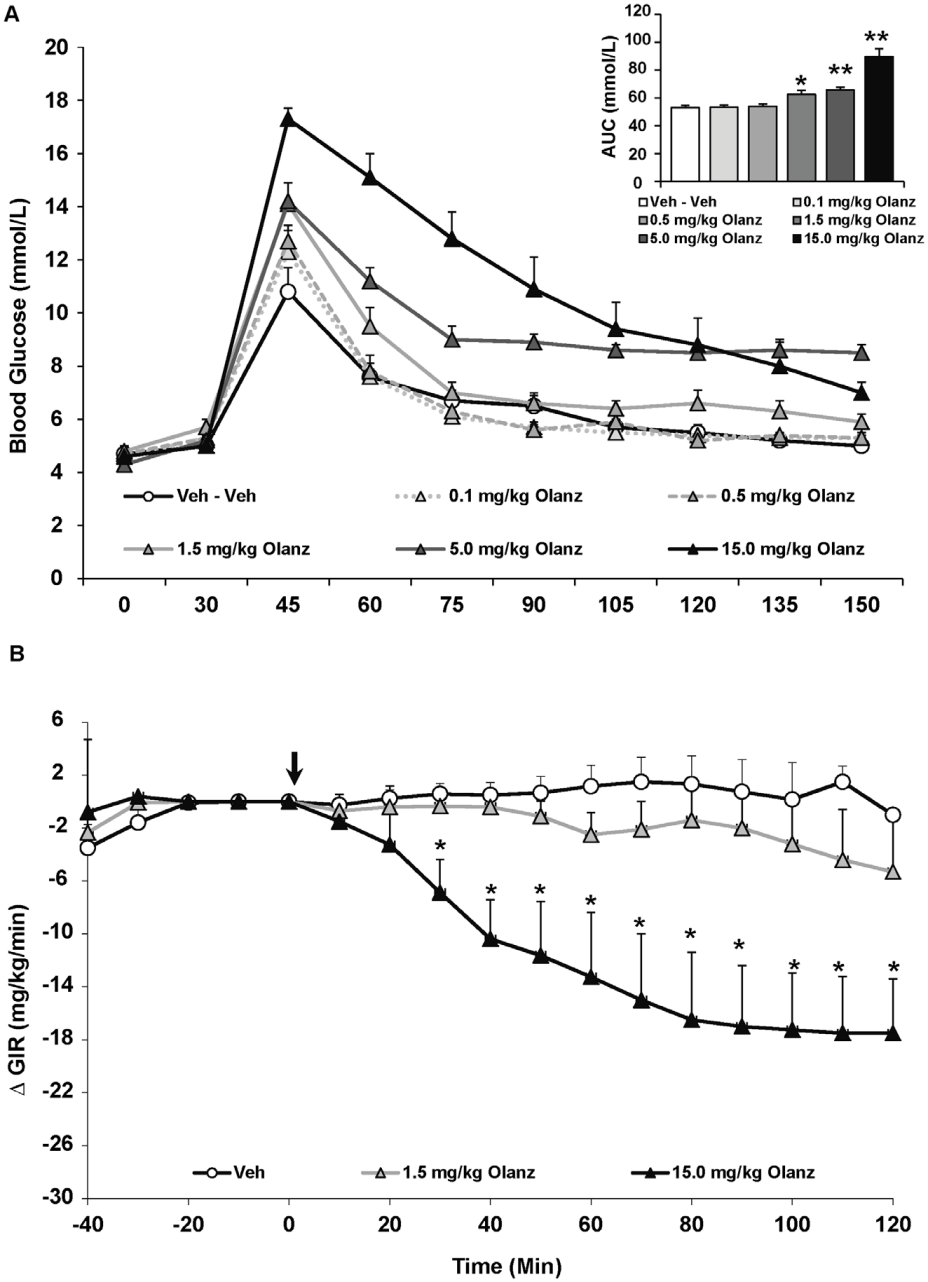
Acute effects of the atypical antipsychotic drug olanzapine on glucose levels in adult female rats. (**A**) Animals (n = 8–10 per group) received a single injection of vehicle or olanzapine (0.1, 0.5, 1.5, 5.0, 15.0 mg/kg, s.c). Glucose levels were recorded prior to drug treatment in overnight-fasted rats at Time 0, and then 30 minutes following drug administration (*x-axis*). Immediately following this glucose measurement, all rats were subjected to a glucose tolerance test by receiving an intraperitoneal challenge injection of 1 mg/ml/kg of glucose, and blood glucose levels were measured every 15 minutes for the next two hours. Total cumulative glucose levels for each treatment group are summed as the “area under the curve” during the glucose tolerance test by graph insets (*top right*). Values represent group means ± SEM. * indicates different from vehicle-treated animals, p<0.05; ** indicates different from vehicle-treated animals, p<0.01 (**B**). A separate cohort of animals (n = 6–8 per group) were fasted over-night and subjected to the hyperinsulinemic-euglycemic clamp. After animals reached euglycemia (three consecutive blood glucose readings of 6.0±0.4 mmol/L), rats were treated with vehicle, low (1.5 mg/kg) or high dose (15.0 mg/kg) olanzapine (arrow at t = 0 min). Glucose levels were recorded every 10 minutes and the glucose infusion rate was adjusted as needed. Glucose infusion rates for each treatment group are presented as change in glucose infusion rate from euglycemia. Values represent group means ± SEM. * indicates different from vehicle and low-dose olanzapine-treated animals, p<0.05.

The effects of olanzapine on glucose metabolism were consistent with our previous findings [26], [36]. There was no effect of olanzapine treatment on fasting glucose levels, but a strong effect on glucose tolerance in the IGTT [F_(5,43) = _8.47, p<0.0001] (Figure 4A). There was a dose-dependent effect of olanzapine, whereby the three higher doses increased glucose levels versus controls. This was highly significant for the 5 and 15 mg/kg doses (p<0.001), which increased glucose levels by 24% and 69% compared to controls. Olanzapine also significantly increased insulin levels [F_(5,43) = _2.90, p<0.05] (Table 1). All doses increased insulin levels: this was a trend for the 0.5 and 1.5 mg/kg doses (p = 0.06), while the two highest doses (5 and 10 mg/kg) caused larger increases (p<0.01). The effect of olanzapine on HOMA-IR values was a non-significant trend [F_(5,43) = _2.05, p = 0.09] to increase values. Importantly, values on the washout IGTT given the week after olanzapine treatment did not differ from the baseline IGTT values prior to SGA treatment, indicating that glucose tolerance did not change during the course of the study.

### HIEC

Average basal glucose levels were similar for all groups prior to euglycemia and administration of antipsychotic drugs. Insulin resistance during the HIEC is indicated by a reduction in the GIR, and therefore the primary analysis compared the effects of antipsychotics on GIR.

For the overall ANOVA, specific antipsychotic drug (asenapine, iloperidone, olanzapine or vehicle) and dose (vehicle, lower or higher dose) were represented by between-subjects factors. The results indicated significant main effects of both drug [F_(2,34) = _12.76, p<0.0001], dose [F_(2,34) = _20.78, p<0.0001] and a drug × dose interaction [F_(2,34) = _2.93, p<0.05]. Drug effects were evident as asenapine treatment, regardless of dose, had no effect on the GIR compared to vehicle-treated rats (Figure 2B). By contrast, both iloperidone and olanzapine, regardless of dose, significantly decreased the GIR compared to vehicle-treated rats (p<0.01) (Figure 3B & 4B). Furthermore, both iloperidone (p<0.01) and olanzapine (p<0.05) decreased GIR significantly more than asenapine. For iloperidone, both doses (1.0 and 10.0 mg/kg) caused significant decreases in the GIR. In both iloperidone dose groups this was evident by 30 mins post treatment, and by 40 mins the higher-dose group had a significantly lower GIR than the lower-dose group. In the olanzapine group, only the higher dose of the drug (15 mg/kg) significantly decreased infusion rate throughout the duration of the HIEC (p<0.01). The higher dose olanzapine group showed significantly decreased GIR compared to both controls and lower-dose animals within 30 mins of treatment. While the magnitude of the decrease in GIR was greater with iloperidone than olanzapine, this effect did not quite achieve statistical significance.

## Discussion

In the present study, we assessed the metabolic side-effects of the novel SGA drugs asenapine and iloperidone for the first time, as well as olanzapine for reference. Two separate techniques were used to measure metabolic dysregulation, including both the glucose tolerance test and the hyperinsulinemic-euglycemic clamp. Consistent with previous preclinical studies, olanzapine caused dose-dependent glucose intolerance in the IGTT and insulin resistance in the clamp [26], [27], [40]–[47]. By contrast, the novel SGA drug asenapine was largely devoid of metabolic effects at the doses tested, causing only a slight reduction in insulin levels and HOMA-IR values with the lowest dose. In comparison, the novel SGA drug iloperidone showed potent dose-dependent effects on metabolic function. The three highest doses in the IGTT substantially increased glucose intolerance, to a greater degree than that observed with olanzapine. Similarly, both low- and high-dose iloperidone increased insulin resistance in the euglycemic clamp.

These overall findings reconfirm the powerful effect of the SGA olanzapine in animal models of glucose control and uptake, and demonstrate for the first time that similar-to-greater magnitude effects were observed with iloperidone, while asenapine shows minimal metabolic liability. The glucose tolerance test assesses glucose “intolerance” by measuring the capacity of the fasted subject to restore glucose levels to the normal range over time after a glucose challenge; this procedure is widely used in both clinical and preclinical studies of prediabetes and Type 2 DM [48]. The glucose tolerance test has been viewed positively for its physiological relevance and practicality in measuring metabolic side-effects in animals or humans, as it reflects how glucose will be controlled after a meal [49]. However, as both glucose and insulin levels are free to vary in this test, it is necessary to use “clamp” procedures, such as the hyperinsulinemic-euglycemic clamp, to confirm the presence of whole-body insulin resistance, which provides a specific measure of cell-mediated glucose uptake via the action of insulin. These procedures should be considered as complementary, and the high degree of correlation between them with the current results provides converging evidence for drug-specific metabolic liability. The only exception to this was in rats treated with the 1.5 mg/kg dose of olanzapine, where animals in the IGTT displayed significant glucose intolerance, but the same dose did not cause significant insulin resistance in the clamp. It is therefore possible that the IGTT is more sensitive at detecting SGA-induced metabolic dysregulation.

As noted above, numerous studies have previously reported metabolic dysregulation following both acute and chronic treatment with olanzapine. We recently reported that rats treated daily with olanzapine for ten weeks showed no change in the magnitude of glucose intolerance in the IGTT compared to their first challenge with the drug [36], indicating that acute treatment with SGAs can model chronic drug-treatment effects. However, to our knowledge, there are no studies of glucose intolerance or insulin resistance with the SGAs asenapine and iloperidone, in either animals or humans. Clinically, in three short-term phase 3 trials of iloperidone for schizophrenia, non-fasting glucose levels in all three dose ranges were significantly increased compared to placebo-treated subjects, whereas the risperidone comparator group did not differ from placebo [50]. Both risperidone- and iloperidone-treated subjects exhibited significant weight-gain versus placebo. A separate 28 day clinical trial of iloperidone in head-to-head comparison with ziprasidone noted greater weight-gain in the iloperidone arm [51], with 21% of iloperidone subjects (versus 7% of ziprasidone and 3% of placebo) displaying clinically significant weight-gain. Changes in glucose levels, which were not specified with regards to fasting status, were 7.9 mg/dL for iloperidone versus 4.7 mg/dL for ziprasidone. An analysis of three long-term safety trials of iloperidone, with haloperidol as comparator, noted both greater weight-gain and increases in glucose levels at six weeks and 52 weeks following treatment in the iloperidone group [52]. Thus, clinical data suggest that both fasting and non-fasting glucose levels are increased by iloperidone, compared to other antipsychotic drugs including risperidone, ziprasidone and haloperidol, none of which may be considered high metabolic risk. However, data from preclinical studies with SGAs indicate that fasting levels of glucose in the absence of a glucose challenge may underestimate loss of glycemic control. For example, in the present study, fasting glucose levels were not increased by any of the SGAs, yet for two drugs severe glucose intolerance was observed when the glucose challenge was applied.

There are a larger number of studies that have reported the effects of asenapine on weight-gain and fasting glucose levels, in both schizophrenia and bipolar disorder, although a full summary of these findings is beyond the scope of the present discussion. Asenapine causes less weight-gain and glucose elevation than olanzapine [53]–[55]. When compared head-to-head against haloperidol, neither drug caused significant weight-gain versus placebo, and fasting glucose abnormalities were actually marginally lower in the two doses of asenapine than in the haloperidol-treated group [56]. A short term study of asenapine only, with no comparator, for psychosis in the elderly reported a non-significant decrease in the number of subjects meeting criteria for metabolic syndrome compared to baseline [57]. Clinical data therefore indicate minimal effects of asenapine on weight-gain and fasting glucose. Similarly, preclinical evidence indicates that the antipsychotic drug sulpiride may also have negligible effects on glycemic control and may even improve glucose clearance in female rats. Compared to both control and risperidone-treated animals, sulpiride administration was associated with a 13% reduction in the area under the curve for the GTT, despite increased body weight gain [33].

A potential issue regarding the current findings is the choice of SGA doses, as metabolic effects can be dose-dependent [58]. Clinical studies can compare metabolic side-effects between antipsychotic drugs at doses of equivalent clinical efficacy, using standardized measures such as chlorpromazine equivalency [59]–[61]. This is not possible in animal models, and so “head-to-head” comparison between drugs represents a theoretical challenge, despite its common practice. It has been suggested that dosing based on dopamine D_2_ receptor occupancy may represent one strategy, but issues remain regarding route of administration and inconsistent effects for all SGAs [62]. We chose our current dosing based on the behavioral effects of drugs in preclinical screens and models of schizophrenia. Behavioral effects in the latter share some homology with clinical symptomatology in humans [63]. Previous behavioral paradigms with asenapine in rats have shown potent antipsychotic-like effects below a dose of 0.2 mg/kg. Franberg and colleagues reported improvement in the conditioned avoidance response task with doses from 0.05–0.2 mg/kg [64] while doses from 0.01–0.075 mg/kg reversed phencyclidine (PCP)-induced deficits in a novel object recognition task [65]. Marston and colleagues demonstrated that 0.03 mg/kg asenapine reversed low-dose amphetamine hyperactivity, while 0.1 mg/kg reversed high-dose amphetamine hyperactivity [66]. In the same study, 0.03–0.1 mg/kg asenapine reversed apomorphine-induced deficits in prepulse inhibition (PPI) of the acoustic startle reflex. Thus, the 100-fold dose range in the current study more than encompasses the dosing required to produce behavioral effects, and the total absence of metabolic effects at doses as high as 0.5 and 1.0 mg/kg strongly indicates the metabolic liability of asenapine is low at behaviorally relevant doses. Regarding iloperidone, an initial report observed behavioral effects at doses between 0.7–5.2 mg/kg [67] while Barr and colleagues reported that both 1.0 and 3.0 mg/kg iloperidone reversed apomorphine-induced PPI deficits [68]. However, 3.0 mg/kg of iloperidone did not reverse the effects on PPI of 1.5 mg/kg of PCP, which is a dose commonly used to screen for antipsychotics, indicating that higher doses of iloperidone would have been needed to demonstrate antipsychotic efficacy. By comparison, 10 mg/kg olanzapine robustly reversed PPI deficits caused by 1.5 mg/kg PCP [69], implying that both iloperidone and olanzapine may be reasonably well-matched dose-wise in the current study. The clinically approved daily dose of iloperidone for psychosis is 12–24 mg [32], while olanzapine is 10–20 mg [70] and therefore very similar. Thus, the greater glucose intolerance and insulin resistance caused by iloperidone versus olanzapine at similar doses (e.g. 5 mg/kg) indicates that iloperidone may have acute metabolic effects at least as strong as olanzapine.

The results of the current study strongly suggest that metabolic testing of patients treated with novel SGAs is warranted. Although, like numerous preclinical studies, the current results were based on acute treatment without weight-gain, there is an increasing body of evidence demonstrating weight-independent and drug-specific effects on glucose intolerance and insulin resistance. Results from the Clinical Antipsychotic Trial of Intervention Effectiveness (CATIE) study observed that 42.7% of patients treated with SGAs had metabolic dysregulation [71]. When controlling for Body Mass Index, CATIE men were 85% and CATIE women 137% more likely to have metabolic syndrome than the normal population. Newcomer and colleagues used the glucose tolerance test in adiposity-matched patients to show that those treated with SGAs had greater glucose elevations than patients treated with first generation drugs [72]. Importantly, in the first double-blind, placebo-controlled crossover trial, it was recently demonstrated that acute 3-day treatment of normals with 10 mg of olanzapine caused significant impairments in the glucose tolerance test [18]. The glucose area-under-the-curve increased by 42%, which is highly consistent with our present findings in rats. Future clinical evaluation of glucose intolerance and insulin resistance in novel SGA-treated patients should therefore remain a priority, using techniques specifically designed to challenge metabolic regulation, despite the greater difficulty of using such protocols, given the long-term health implications for loss of glycemic control.
